# Prognostic value of PAM50 and risk of recurrence score in patients with early-stage breast cancer with long-term follow-up

**DOI:** 10.1186/s13058-017-0911-9

**Published:** 2017-11-14

**Authors:** Hege O. Ohnstad, Elin Borgen, Ragnhild S. Falk, Tonje G. Lien, Marit Aaserud, My Anh T. Sveli, Jon A. Kyte, Vessela N. Kristensen, Gry A. Geitvik, Ellen Schlichting, Erik A. Wist, Therese Sørlie, Hege G. Russnes, Bjørn Naume

**Affiliations:** 1 Division of Cancer Medicine, Department of Oncology, Oslo University Hospital, Postbox 4953 Nydalen, 0424 Oslo, Norway; 2 Division of Laboratory Medicine, Department of Pathology, Oslo University Hospital, Oslo, Norway; 3 Oslo Centre for Biostatistics and Epidemiology, Oslo University Hospital, Oslo, Norway; 4 Department of Cancer Genetics, Institute of Cancer Research, Oslo University Hospital, Oslo, Norway; 5 Division of Medicine, Department of Clinical Molecular Biology, Akershus University Hospital, Lørenskog, Norway; 6 Breast and Endocrine Surgery Unit, Division of Cancer Medicine, Department of Oncology, Oslo University Hospital, Oslo, Norway; 7 Institute of Clinical Medicine, University of Oslo, Oslo, Norway

**Keywords:** Breast cancer, PAM50, Risk of recurrence, Patient stratification, Follow-up

## Abstract

**Background:**

The aim of this study was to investigate the prognostic value of the PAM50 intrinsic subtypes and risk of recurrence (ROR) score in patients with early breast cancer and long-term follow-up. A special focus was placed on hormone receptor-positive/human epidermal growth factor receptor 2-negative (HR+/HER2−) pN0 patients not treated with chemotherapy.

**Methods:**

Patients with early breast cancer (*n* = 653) enrolled in the observational Oslo1 study (1995–1998) were followed for distant recurrence and breast cancer death. Clinicopathological parameters were collected from hospital records. The primary tumors were analyzed using the Prosigna® PAM50 assay to determine the prognostic value of the intrinsic subtypes and ROR score in comparison with pathological characteristics. The primary endpoints were distant disease-free survival (DDFS) and breast cancer-specific survival (BCSS).

**Results:**

Of 653 tumors, 52.2% were classified as luminal A, 26.5% as luminal B, 10.6% as HER2-enriched, and 10.7% as basal-like. Among the HR+/HER2− patients (*n* = 476), 37.8% were categorized as low risk by ROR score, 22.7% as intermediate risk, and 39.5% as high risk. Median follow-up durations for BCSS and DDFS were 16.6 and 7.1 years, respectively. Multivariate analysis showed that intrinsic subtypes (all patients) and ROR risk classification (HR+/HER2− patients) yielded strong prognostic information. Among the HR+/HER2− pN0 patients with no adjuvant treatment (*n* = 231), 53.7% of patients had a low ROR, and their prognosis at 15 years was excellent (15-year BCSS 96.3%). Patients with intermediate risk had reduced survival compared with those with low risk (*p* = 0.005). In contrast, no difference in survival between the low- and intermediate-risk groups was seen for HR+/HER2− pN0 patients who received tamoxifen only. Ki-67 protein, grade, and ROR score were analyzed in the unselected, untreated pT1pN0 HR+/HER2− population (*n* = 171). In multivariate analysis, ROR score outperformed both Ki-67 and grade. Furthermore, 55% of patients who according to the PREDICT tool (http://www.predict.nhs.uk/) would be considered chemotherapy candidates were ROR low risk (33%) or luminal A ROR intermediate risk (22%).

**Conclusions:**

The PAM50 intrinsic subtype classification and ROR score improve classification of patients with breast cancer into prognostic groups, allowing for a more precise identification of future recurrence risk and providing an improved basis for adjuvant treatment decisions. Node-negative patients with low ROR scores had an excellent outcome at 15 years even in the absence of adjuvant therapy.

**Electronic supplementary material:**

The online version of this article (doi:10.1186/s13058-017-0911-9) contains supplementary material, which is available to authorized users.

## Background

Breast cancer survival has improved during the last two decades because of both early detection and improved treatment strategies such as biomarker-defined therapy (i.e., adjuvant endocrine treatment and trastuzumab) along with chemotherapy for high-risk patients. However, the risk of relapse varies substantially on the basis of individual disease [1]. Differences in clinical behavior among patients with early breast cancer were also paralleled at a molecular level, and the “intrinsic” subtypes, later refined into the PAM50 classification, capture biological traits and are recognized as robust subtypes [2, 3]. In line with the increased body of evidence for improved clinical classification using molecular profiling, classifiers such as the PAM50 intrinsic subtypes and risk of recurrence (ROR) score generated from the expression of the 50 genes (Prosigna®; NanoString Technologies, Seattle, WA, USA) have recently been included in recommendations for decisions on adjuvant systemic treatment for pN0 hormone receptor-positive/human epidermal growth factor receptor 2-negative (HR+/HER2−) breast cancer [4, 5]. Several studies have also emphasized the impact of PAM50 subtypes and ROR scores in assessment of late distant recurrence after endocrine treatment [6, 7]. A number of gene assays have been developed to predict outcomes beyond standard clinicopathological variables, two of which (Oncotype DX, Genomic Health, Redwood City, CA, USA; and MammaPrint, Agendia, Irvine, CA, USA) are currently being evaluated in large, prospective, randomized trials. Results after 5 years with endocrine treatment alone show very low rates of recurrence in HR+/HER2−, axillary lymph node-negative (pN0) patients with favorable gene expression [8, 9]. Comparison of multiparameter tests in retrospective analyses, including the prognostic signatures Clinical Treatment Score (CTS), four immunohistochemical markers (IHC4 score), oncotype recurrence score (RS), EndoPredict score (EPclin), Breast Cancer Index (BCI), and ROR score [10–16], indicate that EPclin and ROR score may be the strongest predictors of distant recurrence in both node-positive and node-negative HR+ patients with breast cancer. Particularly, EPclin and ROR score appear to be promising identifiers of patients at low risk for distant recurrence, with a potential to outperform CTS [17]. Hence, these classifiers may identify patients who may be spared adjuvant chemotherapy and be sufficiently treated with endocrine treatment only, unlike those classified as having a high risk of relapse.

The use of molecular profiling has not yet been widely established in all countries, and additional studies may provide important information regarding long-term survival and how to include the tests in clinical routine. The aim of the present study was to evaluate the long-term prognostic value of the PAM50 intrinsic subtypes, and especially the ROR score, in patients with HR+/HER2− early-stage breast cancer after extended follow-up.

## Methods

### Patients and tumor characteristics

**Fig. 1:**
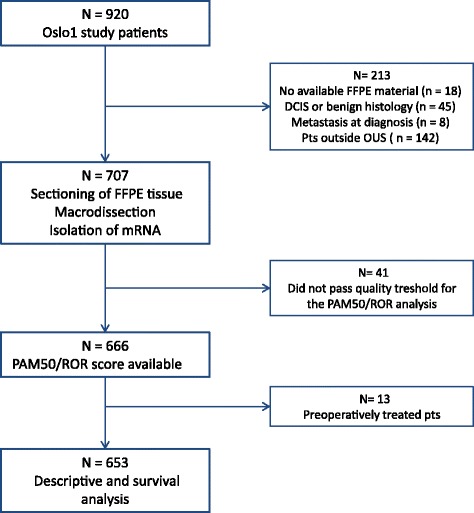
Overview of patients included in the analysis. *DCIS* Ductal carcinoma in situ, *FFPE* Formalin-fixed, paraffin-embedded, *mRNA* Messenger RNA, *Oslo1* Oslo Micrometastasis Project, *OUS* Oslo University Hospital, *ROR* Risk of recurrence

Consecutive patients with early breast cancer from the observational Oslo Micrometastasis Project (the Oslo1 study) (*n* = 920) who were enrolled at Oslo University Hospital (*n* = 778) and for whom there were available formalin-fixed, paraffin-embedded (FFPE) samples from the primary tumor (*n* = 760) were included in the present study (Fig. 1). Patients with tumors determined to be benign or with in situ histology (*n* = 45) were excluded, as were patients who received neoadjuvant treatment (*n* = 13) or had metastasis at diagnosis (*n* = 8). Furthermore, 41 patients were excluded because their samples did not pass the quality threshold for the PAM50/ROR analysis. The patients were included from 1995 to 1998 and were all treated as per national recommendations. At the time of enrollment, patients with pT1pN0 (regardless of grade) and pT2pN0 grade 1 received no adjuvant treatment (neither tamoxifen [if HR+] nor chemotherapy), whereas those with pT2pN0G2-3, pT3-4pN0 (regardless of grade) or pN1-3 received systemic treatment (i.e., tamoxifen and/or chemotherapy), except for HR− patients aged ≥ 65 years. Chemotherapy (CMF regimen, which consisted of six cycles [every 3 weeks] of intravenous cyclophosphamide 600 mg/m^2^, methotrexate 40 mg/m^2^, and fluorouracil 600 mg/m^2^) was administered if patients were < 55 years of age or aged ≤ 65 years with HR− tumors. Patients with HR+ disease received tamoxifen for 5 years.

Clinical and histopathological parameters were collected from hospital records. HR was defined as positive if ≥ 10% of the cells were stained positive by estrogen receptor (ER) and/or progesterone receptor (PgR). Amplification of the *HER2* gene was assessed as previously described [18]. Ki-67 labeling index (as hot spot) was determined (retrospectively) in the pT1pN0 patients as described in Additional file 1.

Information on follow-up and vital status was obtained through review of medical records and data from the Norwegian death cause registry (provided by the Norwegian Institute of Public Health). We considered a death related to breast cancer when breast cancer was the underlying cause of death. The last obtainable update of disease relapse was completed in 2005. The follow-up for breast cancer death was completed on December 31, 2014.

### PAM50 assay description and ROR calculation

RNA was extracted (High Pure FFPET RNA Isolation Kit, catalogue number 06650775001; Roche Applied Science, Penzberg, Germany) from FFPE blocks of breast cancer tumor tissue, and expression of the PAM50 genes was analyzed using the nCounter Analysis System (NanoString Technologies). Data were analyzed using the Prosigna® algorithm (NanoString Technologies) converted into intrinsic subtype calls, ROR scores, and risk categories as previously described [19, 20]. Tumors with ROR scores ≤ 40 were categorized as low ROR, 41–60 as intermediate risk if pN0 and high risk if pN1, and > 60 as high risk. All patients with pN2-3 were categorized as high risk, regardless of ROR score.

### Statistics

Descriptive statistics were used for patients and tumor characteristics, and results are presented as frequencies and proportions. The patients studied were selected from an observational study with distant disease-free survival (DDFS) and breast cancer-specific survival (BCSS) as primary outcomes [21]. DDFS was defined as time from surgery to any distant metastasis, and BCSS was defined as time from surgery to breast cancer-related death. The patients were followed longitudinally from the date of surgery to the date of distant metastasis or date of death, or to the end of follow-up if no event had occurred. In the survival analyses, patients were censored at 15-year follow-up for breast cancer death and 8-year follow-up for distant metastasis (80% of maximal follow-up time). Survival was presented in Kaplan-Meier plots based on log-rank tests. Analyses were performed across all patients, as well as according to subgroups by administration of systemic treatment, HR/HER2 status, PAM50 intrinsic subtypes, and ROR score risk categories.

Uni- and multivariate Cox regression models were conducted to evaluate the impact of the prognostic factors on DDFS and BCSS. Risk estimates are presented as hazard ratios with 95% CI. The assumption of proportional hazards was met on the basis of analysis of Schoenfeld residuals. No multicollinearity between the independent variables (tested by variance inflation factor analysis) was found.

All *p* values were two-tailed, and *p* < 0.05 was regarded as significant. However, owing to the large number of subgroup analyses, the significance level of the log-rank tests was set to *p* < 0.005. Data analysis was performed using Stata version 14 software (StataCorp, College Station, TX, USA).

## Results

### Patient/tumor characteristics and PAM50 subtype/ROR score

An overview of the selection of patients included in the present study (*n* = 653) is presented in Fig. 1. Patient characteristics are listed in Table 1. Median age at inclusion was 57.7 years (range 27.5–93.0). A total of 331 patients (50.7%) received no adjuvant treatment, 164 (25.1%) received tamoxifen only, and 158 (24.2%) received CMF with or without tamoxifen. By PAM50 gene expression profiling, tumors were classified into subtypes as luminal A (52.2%), luminal B (26.5%), HER2-enriched (10.6%), and basal-like (10.7%). Among the HR+/HER2− patients, 37.8% were categorized as low risk by ROR score, 22.7% as intermediate risk, and 39.5% as high risk (Table 2). As expected, luminal A subtype and low ROR score were more frequent among the node-negative than among the node-positive patients. Moreover, the majority of the tumors categorized as HR+/HER2− by immunohistochemistry were of the luminal A or B subtype (94%) (Additional file 1: Figure S1). Finally, luminal A tumors showed markedly lower ROR scores than the other three subtypes (Additional file 1: Figure S2).

**Table 1:** Baseline characteristics

	No. of patients	Percent
All patients	653	100
Age at inclusion		
≥ 55 years	271	41.5
< 55 years	382	58.5
T status		
pT1	377	57.8
pT2	234	35.8
pT3-4	26	4.0
pTX	16	2.5
N status		
pN0	419	64.2
pN1	136	20.8
pN2	59	9.0
pN3	23	3.5
pNX	16	2.5
Histological grade		
I	153	23.4
II	322	49.3
III	177	27.1
Missing	1	0.2
HR status		
Positive (≥ 10%)	512	78.4
Negative (0 to < 10%)	137	21.0
Missing	4	0.6
HER2 status		
Negative	578	88.5
Positive	71	10.9
Missing	4	0.6
HR/HER2 subclasses		
HR+/HER2−	476	72.9
HR+/HER2+	36	5.5
HR−/HER2+	35	5.4
HR−/HER2−	102	15.6
Missing	4	0.6
Ki-67 (*n* = 218)^a^		
< 15%	103	47.2
15–30%	77	35.3
≥ 30%	38	17.4
Histological subtype		
Ductal	499	76.4
Lobular	121	18.5
Other	33	5.1
Adjuvant treatment		
No adjuvant	331	50.7
Tamoxifen only	164	25.1
CMF with or without Tam	158	24.2

*Abbreviations: CMF* Cyclophosphamide, methotrexate, fluorouracil, *HER2* Human epidermal growth factor receptor 2, *HR* Hormone receptor, *TAM* Tamoxifen

^a^Ki-67 analysis (hot spot) of the pT1pN0 patients, 171 of whom were HR+/HER2−

**Table 2:** Frequency distribution of PAM50 intrinsic subtypes and risk of recurrence score

	All patients		Node-negative patients^a^		Node-positive patients^a^	
	No. of patients	%	No. of patients	%	No. of patients	%
All patients	653	100	419	64.2	218	33.4
PAM50 intrinsic subtype						
Luminal A	341	52.2	240	57.3	95	43.6
Luminal B	173	26.5	103	24.6	65	29.8
HER2enriched	69	10.6	30	7.2	38	17.4
Basal-like	70	10.7	46	11.0	20	9.2
ROR score, median	51		46		56	
HR+/HER2− patients	476	100	318	66.8	149	31.3
ROR risk score classification						
Low risk	180	37.8	145	45.6	33	22.1
Intermediate risk	108	22.7	104	32.7	–	–
High risk	188	39.5	69	21.7	116	77.9
ROR score, median	46		42		50	

*Abbreviations: HER2* Human epidermal growth factor receptor 2, *HR* Hormone receptor, *ROR* Risk of recurrence

^a^Nine patients were classified as pNX

### PAM50 subtype/ROR score classification and clinical outcome

The median follow-up times for BCSS and DDFS were 16.6 years (range 0.4–19.7) and 7.1 years (0.1–10.4), respectively. Overall, 164 patients (25.1%) died of breast cancer. The 5- and 10-year DDFS rates were 83.8% (95% CI 80.6–86.5%) and 74.2% (95% CI 69.3–78.4), respectively, and the 10- and 15-year BCSSs were 79.6% (95% CI 76.2–82.5%) and 75.0% (95% CI 71.3–78.3%).

**Fig. 2:**
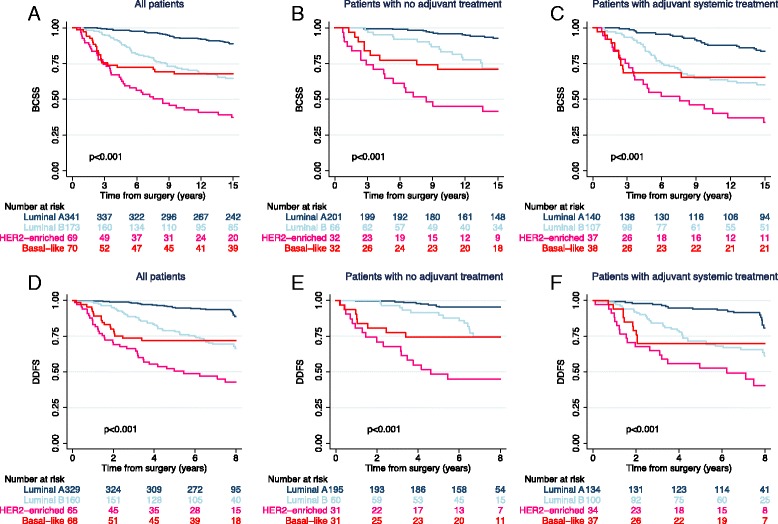
Kaplan-Meier plots of breast cancer-specific survival (BCSS) (**a–c**) and distant disease-free survival DDFS (**d–f**) according to PAM50 subtypes in all 653 patients (**a**, **d**), patients with no adjuvant treatment (**b**, **e**), and patients with adjuvant treatment (**c**, **f**). *p* Values were derived from log-rank tests. *HER2* Human epidermal growth factor receptor 2

Survival analyses according to PAM50 subtypes showed that patients with the luminal A subtype had favorable BCSS and DDFS, particularly in the subgroup of patients who did not receive adjuvant systemic treatment (Fig. 2). The HER2-enriched patients (who did not receive HER2-directed treatment) had the worst outcomes. The PAM50 subtype classification also separated and refined clinical outcomes for patients within HR/HER2 subgroups (Additional file 1: Figure S3). Multivariate analysis confirmed the prognostic impact of this classification, showing that patients with luminal A tumors had improved BCSS and DDFS compared with patients with the other subtypes (Additional file 2: Table S1).

**Fig. 3:**
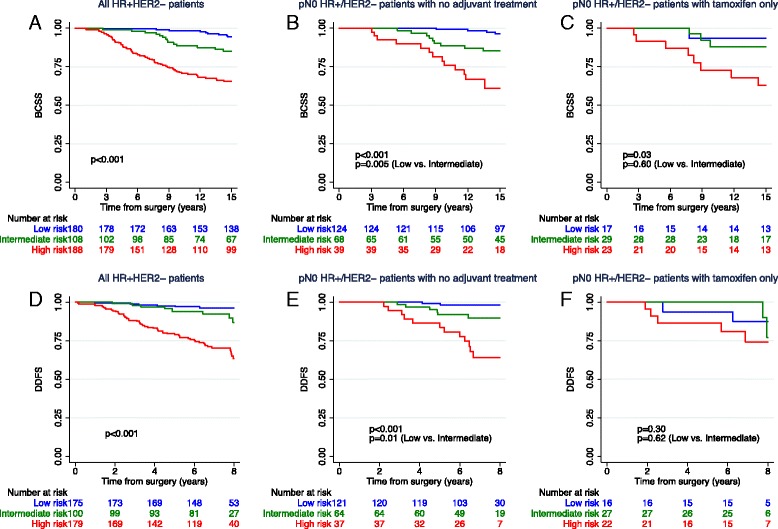
Kaplan-Meier plots of breast cancer-specific survival (BCSS) (**a–c**) and distant disease-free survival (DDFS) (**d–f**) according to risk of recurrence categories within the pN0 hormone receptor-positive/human epidermal growth factor receptor 2-negative (HR+/HER2−) subgroup for all patients (**a**, **d**), patients with no adjuvant treatment (**b**, **e**), and patients with adjuvant tamoxifen only (**c**, **f**). *p* Values were derived from log-rank tests

Among the HR+/HER2− patients, the ROR risk classification separated patients with different BCSS and DDFS survival (*see* Fig. 3 and Additional file 1: Figure S4 for separate analysis of node-negative and node-positive patients). In multivariate analysis accounting for pT, pN, grade, age, and systemic treatment, ROR score was an independent prognostic factor (Table 3). Patients classified as ROR high risk had a markedly increased risk of breast cancer death and distant metastasis compared with patients with ROR low risk (hazard ratio 4.69, 95% CI 2.08–10.55; and hazard ratio 6.82, 95% CI 2.62–17.81, respectively) (Table 3). The hazard ratio for BCSS among patients with intermediate risk versus low risk was 2.25 (95% CI 0.94–5.41, *p* = 0.070).

**Table 3:** Multivariate analysis of distant disease-free survival and breast cancer-specific survival for the HR+/HER2− patients, including risk of recurrence risk classification

	DDFS (*n* = 441)			BCSS (*n* = 461)		
	Hazard ratio	95% CI	*p* Value	Hazard ratio	95% CI	*p* Value
pT						
1	1			1		
2	1.70	0.97–3.00	0.065	1.52	0.91–2.52	0.107
3-4	2.30	0.86–6.14	0.097	2.41	0.99–4.21	0.054
Grade						
I	1			1		
II	1.47	0.69–3.13	0.319	1.68	0.82–3.42	0.156
III	1.39	0.56–3.42	0.474	2.35	1.04–5.35	0.041
pN						
0	1			1		
1	1.31	0.61–2.81	0.492	1.22	0.61–2.45	0.578
2-3	2.27	1.04–4.98	0.040	2.04	0.99–4.21	0.054
Age						
< 55 years	1			1		
≥ 55 years	0.58	0.30–1.11	0.102	0.63	0.36–1.14	0.130
Systemic treatment						
No	1			1		
Tam	0.99	0.48–2.05	0.982	1.06	0.56–2.03	0.854
Chemotherapy ± Tam	0.40	0.16–1.02	0.055	0.48	0.21–1.10	0.084
ROR score						
Low	1			1		
Intermediate	2.25	0.77–6.60	0.137	2.25	0.94–5.41	0.070
High	6.82	2.62–17.81	< 0.001	4.69	2.08–10.55	< 0.001

*Abbreviations: BCSS* Breast cancer-specific survival, *DDFS* Distant disease-free survival, *HER2* Human epidermal growth factor receptor 2, *HR* Hormone receptor, *ROR* Risk of recurrence, *TAM* Tamoxifen

### ROR score and clinical outcome within the node negative HR+/HER2− subgroups

Because only 18 (5.7%) of the 318 node-negative HR+/HER2− patients received chemotherapy and 231 (72.6%) received neither chemotherapy nor endocrine treatment, this subgroup of patients was studied in more detail to unveil the impact of the ROR categories on long-term risk of distant disease and breast cancer death without use of chemotherapy with or without endocrine treatment. Among those who did not receive any adjuvant treatment (*n = 231*), 53.7%, 29.4%, and 16.9% of the patients were assigned to the low-, intermediate-, and high-risk ROR risk groups, respectively. Patients with low ROR risk had an excellent prognosis at 15 years (15-year BCSS 96.3%, 95% CI 90.4–98.6%), whereas the intermediate- and high-risk groups had reduced survival (BCSS 85.2%, 95% CI 73.4–92.0%; and 60.8%, 95% CI 42.8–74.7%, respectively) (Fig. 3). There was a difference in BCSS for patients in ROR low-risk versus ROR intermediate-risk categories (*p* = 0.005). In contrast, among patients who received tamoxifen only (*n* = 69), no difference in survival between the low- and intermediate-risk groups was observed (*p* = 0.60). Similar results were found when the analysis was restricted to the luminal A pN0 HR+/HER2− patients (Additional file 1: Figure S5). Both the untreated and tamoxifen-treated, node-negative, HR+/HER2− patients classified as ROR high risk appeared to have reduced survival (Fig. 3).

### ROR versus Ki-67 analysis in node-negative HR+/HER2− pT1 subgroup

The Ki-67 labeling indexes (hot spot) were previously determined for the patients with HR+/HER2− pT1pN0 tumors, representing an unselected subgroup of untreated patients (who were not recommended any adjuvant treatment at the time of inclusion) [18]. The correlation between Ki-67 expression and ROR score for the HR+/HER2− tumors is shown in Additional file 1: Figure S6. Although the observed correlation was fair, the degree of variability poses a challenge in applying strict cutoff values. Multivariate analysis of BCSS and DDFS, including ROR score, Ki-67 (as both continuous and categorical variables), and histologic grade, revealed that only ROR score remained a significant prognostic factor (Table 4).

**Table 4:** Multivariate analysis of distant disease-free survival for the HR+/HER2− pT1pN0 patients, including Ki-67 and risk of recurrence score as continuous and categorical variables

	DDFS (*n* = 164)			BCSS (*n* = 171)		
	Hazard ratio	95% CI	*p* Value	Hazard ratio	95% CI	*p* Value
Ki-67 and ROR score as continuous variables						
Age						
< 55 years	1			1		
≥ 55 years	1.71	0.50–5.80	0.391	1.10	0.41–3.00	0.849
Grade						
I	1			1		
II	1.56	0.31–7.99	0.591	1.87	0.48–7.21	0.366
III	1.26	0.16–9.86	0.827	2.25	0.40–12.61	0.355
Ki-67	1.04	0.98–1.09	0.172	1.01	0.96–1.06	0.729
ROR score	1.05	1.01–1.09	0.011	1.05	1.02–1.08	0.002
Ki-67 and ROR score as categorical variables						
Age						
< 55 years	1			1		
≥ 55 years	1.36	0.44–4.23	0.598	1.06	0.41–2.72	0.912
Grade						
I	1			1		
II	1.88	0.34–10.34	0.468	2.19	0.56–8.55	0.258
III	1.70	0.22–13.01	0.609	2.62	0.47–14.50	0.271
Ki-67						
< 15%	1			1		
15–30%	0.62	0.12–3.31	0.576	0.66	0.19–2.28	0.514
> 30%	1.94	0.33–11.52	0.465	1.59	0.40–6.34	0.513
ROR score						
Low	1			1		
Intermediate	9.56	1.01–90.87	0.049	4.52	1.08–18.85	0.038
High	27.22	2.46–300.49	0.007	9.09	1.80–14.50	0.008

*Abbreviations: BCSS* Breast cancer-specific survival, *DDFS* Distant disease-free survival, *HER2* Human epidermal growth factor receptor 2, *HR* Hormone receptor, *ROR* Risk of recurrence

### Treatment alteration analysis based on use of ROR classification in node-negative HR+/HER2− pT1 subgroup

We analyzed ROR classification among the 171 HR+/HER2− pT1pN0 patients and compared the results with the estimated benefit of chemotherapy according to the web-based algorithm PREDICT (www.predict.nhs.uk), which is based on standard histopathological criteria. For nine patients, the exact tumor size was missing and thus was excluded from the PREDICT analysis. As presented in Table 5, 33% of the patients who had an absolute chemotherapy benefit ≥ 3% according to PREDICT were classified as ROR low risk. In addition, 22% were classified as luminal A with ROR intermediate risk, and 8% of the patients with < 3% chemotherapy benefit were classified as luminal B with either ROR intermediate or high risk.

**Table 5:** Treatment benefit according to PREDICT model versus risk of recurrence score for patients with HR+/HER2− pT1pN0 disease

	PREDICT model (absolute benefit of chemotherapy, 10-year OS)			
	< 3%	3–5%	≥ 5%	No. of patients
ROR score				
Low	57	30	0	87
Intermediate, Luminal A	8	18	2	16
Intermediate, Luminal B	4	12	3	19
High	2	12	14	28
Total	71	72	19	162

*Abbreviations: HER2* Human epidermal growth factor receptor 2, *HR* Hormone receptor, *OS* Overall survival, *ROR* Risk of recurrence

Ki-67 positivity for the PREDICT model (http://www.predict.nhs.uk/) is defined as > 10% of tumor cells staining positive (average counting). Adjusting for Ki-67 labeling index as a hot spot in our study, Ki-67 positivity for the PREDICT analysis was defined as > 20% of tumor cells staining positive [46]

## Discussion

In this study of patients with early-stage breast cancer with 17 years follow-up, the PAM50 subtypes and ROR scores clearly improved the prognostic classification beyond current clinicopathological parameters. Importantly, we were able to study an unselected subgroup of node-negative patients who did not receive any adjuvant treatment. We identified a large group of patients with node-negative HR+/HER2− disease with an excellent prognosis and questionable benefit of adjuvant chemotherapy. A subgroup of these patients may also have limited advantage of endocrine treatment. The ROR score was superior to histological grade and Ki-67 labeling index as a prognostic factor. In line with other multigene tests, the refinement in risk classification by the ROR score may help the treating physician and the patient arrive at a balanced decision on adjuvant treatment [8, 22–24].

Data are still lacking on the prognostic impact of the ROR score among untreated patients. We were able to study a relatively large group of node-negative HR+/HER2− patients, representing three-fourths of this population, who did not receive any adjuvant systemic treatment (neither tamoxifen nor chemotherapy). About half of these patients were classified in the low ROR risk group and had an excellent long-term prognosis. These results are in line with what was observed in several studies of PAM50 ROR as well as other multigene signatures in HR+/HER2− disease, although the patients in these studies received endocrine treatment [7–9, 12, 13, 15, 25–27]. Together, the available data support omission of chemotherapy to node-negative HR+/HER2− patients with low-risk multiparameter tests.

The recommended use of adjuvant endocrine treatment has changed markedly since the patients were included in the present study. In many guidelines, nearly all HR+ patients are now advised to receive endocrine treatment [4, 5]. However, the patients who did not receive endocrine treatment in our study (any grade pT1pN0 and grade 1 pT2pN0) are comparable to such patients diagnosed today because the median tumor size for the entire study population was identical to what was reported on the national level in Norway in 2015 (17 mm) [28, 29]. The excellent prognosis among patients with node-negative HR+/HER2− disease and low ROR scores in this study indicates that a subgroup of these patients is sufficiently treated without adjuvant endocrine therapy. However, we recognize the limited number of patients in the subgroup analyses. Additional data derived from larger untreated (and unselected) patient series to support these results are warranted but difficult to obtain. Delahaye et al. recently reported an ultralow/indolent signature based on the 70-gene signature, identifying a small subgroup of patients not receiving adjuvant treatment who had 100% 15 years of BCSS [30]. Comparison of this ultralow signature with the low-risk ROR score would be of interest. In the clinic, a significant proportion of patients receiving adjuvant endocrine treatment experience a level of side effects that may challenge the individual benefit of the treatment [31, 32]. Also, poor adherence to/nonpersistence with endocrine treatment has been reported [33]. Because extended adjuvant endocrine treatment recommendations have been introduced to reduce the risk of late recurrence among HR+/HER2− patients, there is also a need for improved selection criteria to better identify candidates for prolonged endocrine treatment [34, 35]. Recent studies have documented the advantage of using multiparameter tests for improved prognostication after 5 years of endocrine treatment, such as EPclin, BCI, and ROR score [14, 36–39]. Our results support the potential use of such tests for extended adjuvant endocrine treatment decisions. New possibilities for improved prognostication of patients who are candidates for endocrine treatment would support the counseling and treatment decisions for the individual patient by taking into account both side effects and recurrence risk (without any endocrine treatment or extended endocrine treatment) [40].

We observed outcomes similar to those with low ROR score (postmenopausal group) for the node-negative HR+/HER2− patients with intermediate ROR scores receiving adjuvant endocrine treatment only. In contrast, patients in the intermediate-risk group had reduced survival compared with those in the low-risk group when no adjuvant treatment was administered. Although this finding should be interpreted with caution owing to the restricted number of patients, this indicates that endocrine treatment without chemotherapy could be a treatment option also for patients with pN0 HR+/HER2− disease with tumors in the intermediate-risk category. In contrast, the results from the ABCSG (Austrian Breast and colorectal Cancer Study Group) and ATAC (Arimidex, Tamoxifen, Alone or in Combination) studies showed reduced DDFS among patients with intermediate-risk ROR scores compared with the low-risk category, all receiving endocrine therapy [14, 25]. However, a definitive answer to the benefit of chemotherapy for these patients needs a randomized clinical trial. The ongoing OPTIMA trial addresses this issue [41].

In addition to the identification of a large low-risk group, the ROR risk classification also sets apart a high-risk group of patients among the node-negative HR+/HER2− subgroup. On the basis of current routine classification, high risk of distant recurrence in a fraction of patients expected to have a relatively low recurrence risk is still a concern [42]. Thus, additional prognostic information by ROR score or other multigene test may help clinicians to better select candidates for chemotherapy, especially in doubtful cases.

On the basis of extrapolated analyses using current treatment recommendations for this retrospective population, adding the ROR information to clinical decision-making may reduce the use of chemotherapy by at least one-third (Table 5). This is in accordance with the results from the EORTC 10041/BIG 3-04 MINDACT (Microarray in Node-Negative and 1 to 3 Positive Lymph Node Disease May Avoid Chemotherapy) trial [8]. Consequently, a reduction in the unnecessary side effects and a reduced health economic burden are expected, but they merit further validation in prospective clinical trials. Recent publications have also suggested that a 13–47% change in treatment decisions will be reflected by future cost-effectiveness analyses [22, 40, 43, 44].

A combination of the biomarkers ER, PgR, HER2, and Ki-67 has been used in treatment guidelines, and these entities act as surrogate markers for the molecular breast cancer subtypes [5]. Despite a focus on standardization of these markers (particularly Ki-67), lack of reproducibility is still a concern [10, 45]. Reports have indicated that the ROR score and intrinsic subtyping are superior to a standardized immunohistochemical classification algorithm (IHC4) [19, 26]. In this study, Ki-67 showed correlation with ROR score, in line with the special weighting of a set of proliferation-associated genes in the ROR score model [26]. Still, the observed variability is large and clearly illustrates that Ki-67 and ROR score are not interchangeable. Importantly, our results show that ROR score outperformed Ki-67 and histological grade as a prognostic factor among the pN0 HR+/HER2− patients. This is also supported by other studies [26].

## Conclusions

Our results support the use of the PAM50 ROR score to improve the classification of patients with breast cancer into prognostic groups, allowing for a more precise identification of future recurrence risks and an improved basis for adjuvant treatment decisions. Patients with node-negative HR+/HER2− tumors with low ROR scores can be treated sufficiently without use of chemotherapy, and some may have such a limited systemic relapse risk that one may question the benefit of adjuvant endocrine treatment in individual cases. Patients with intermediate ROR scores (mainly luminal A patients) may represent an additional subgroup with questionable benefit from chemotherapy, but this merits further studies. The PAM50 and Prosigna® risk classification may result in a significant reduction in the use of adjuvant chemotherapy.

## Additional files

Additional file 1: **Methods.** Immunohistochemical analysis for Ki-67. **Figure S1.** Distribution of PAM50 subtypes within subgroups based on HR and HER2 status. Bars represent percentage of total in each HR/HER2− group. Number is displayed on top of the bar. **Figure S2.** ROR score within each of the PAM50 subtypes for all patients (R statistical software package). **Figure S3.** Kaplan-Meier plots of BCSS (**S3a**) and DDFS (**S3b**) according to HR/HER2 subtypes in all 653 patients (**a**) and according to PAM50 subtypes within different HR/HER2 (**b**–**e**) subgroups. *p* Values were derived from log-rank tests. **Figure S4.** Kaplan-Meier plots of BCSS according to ROR categories for node-negative (**a**) and node-positive (**b**) HR+/HER2− patients. *p* Values were derived from log-rank tests. **Figure S5.** Kaplan-Meier plots of BCSS (**a** and **b**) and DDFS (**c** and **d**) according to ROR categories for node-negative luminal A HR+/HER2− patients with no adjuvant treatment (**a**, **c**) or treated with tamoxifen only (**b**, **d**). *p* Values were derived from log-rank tests. **Figure S6.** Correlation between Ki-67 expression and ROR score for the HR+/HER2− patients. ρ = 0.62, *p* < 0.001 (Pearson correlation). (ZIP 340 kb)

Additional file 2: Table S1. Multivariate analysis of DDFS and BCSS for all patients, including PAM50 intrinsic subtypes. (DOC 49 kb)

## Abbreviations

ABCSG: Austrian Breast and colorectal Cancer Study Group
ATAC: Arimidex, Tamoxifen, Alone or in Combination study
BCI: Breast Cancer Index
BCSS: Breast cancer-specific survival
CMF: Cyclophosphamide, methotrexate, fluorouracil
CTS: Clinical Treatment Score
DCIS: Ductal carcinoma in situ
DDFS: Distant disease-free survival
EORTC 10041/BIG 3-04 MINDACT: Microarray in Node-Negative and 1 to 3 Positive Lymph Node Disease May Avoid Chemotherapy trial
EPclin: EndoPredict score
ER: Estrogen receptor
FFPE: Formalin-fixed, paraffin-embedded
HER2: Human epidermal growth factor receptor 2
HR: Hormone receptor
IHC4 score: Four immunohistochemical markers
mRNA: Messenger RNA
OS: Overall survival
PgR: Progesterone receptor
ROR: Risk of recurrence
RS: Oncotype recurrence score
TAM: Tamoxifen
