# Supplementary material for: Prognostic value of PAM50 and risk of recurrence score in patients with early-stage breast cancer with long-term follow-up
Source: Breast Cancer Res. 2017 Nov 14;19:120. doi: 10.1186/s13058-017-0911-9 (PMC5686844; doi:10.1186/s13058-017-0911-9)
Supplement: Supplementary file 1 — Methods. Immunohistochemical analysis for Ki-67. Figure S1. Distribution of PAM50 subtypes within subgroups based on HR and HER2 status. Bars represent percentage of total in each HR/HER2− group. Number is displayed on top of the bar. Figure S2. ROR score within each of the PAM50 subtypes for all patients (R statistical software package). Figure S3. Kaplan-Meier plots of BCSS (S3a) and DDFS (S3b) according to HR/HER2 subtypes in all 653 patients (a) and according to PAM50 subtypes within different HR/HER2 (b–e) subgroups. p Values were derived from log-rank tests. Figure S4. Kaplan-Meier plots of BCSS according to ROR categories for node-negative (a) and node-positive (b) HR+/HER2− patients. p Values were derived from log-rank tests. Figure S5. Kaplan-Meier plots of BCSS (a and b) and DDFS (c and d) according to ROR categories for node-negative luminal A HR+/HER2− patients with no adjuvant treatment (a, c) or treated with tamoxifen only (b, d). p Values were derived from log-rank tests. Figure S6. Correlation between Ki-67 expression and ROR score for the HR+/HER2− patients. ρ = 0.62, p < 0.001 (Pearson correlation). (ZIP 340 kb) [file 13058_2017_911_MOESM1_ESM.zip › Figure S3a_BCR2.1.pdf]

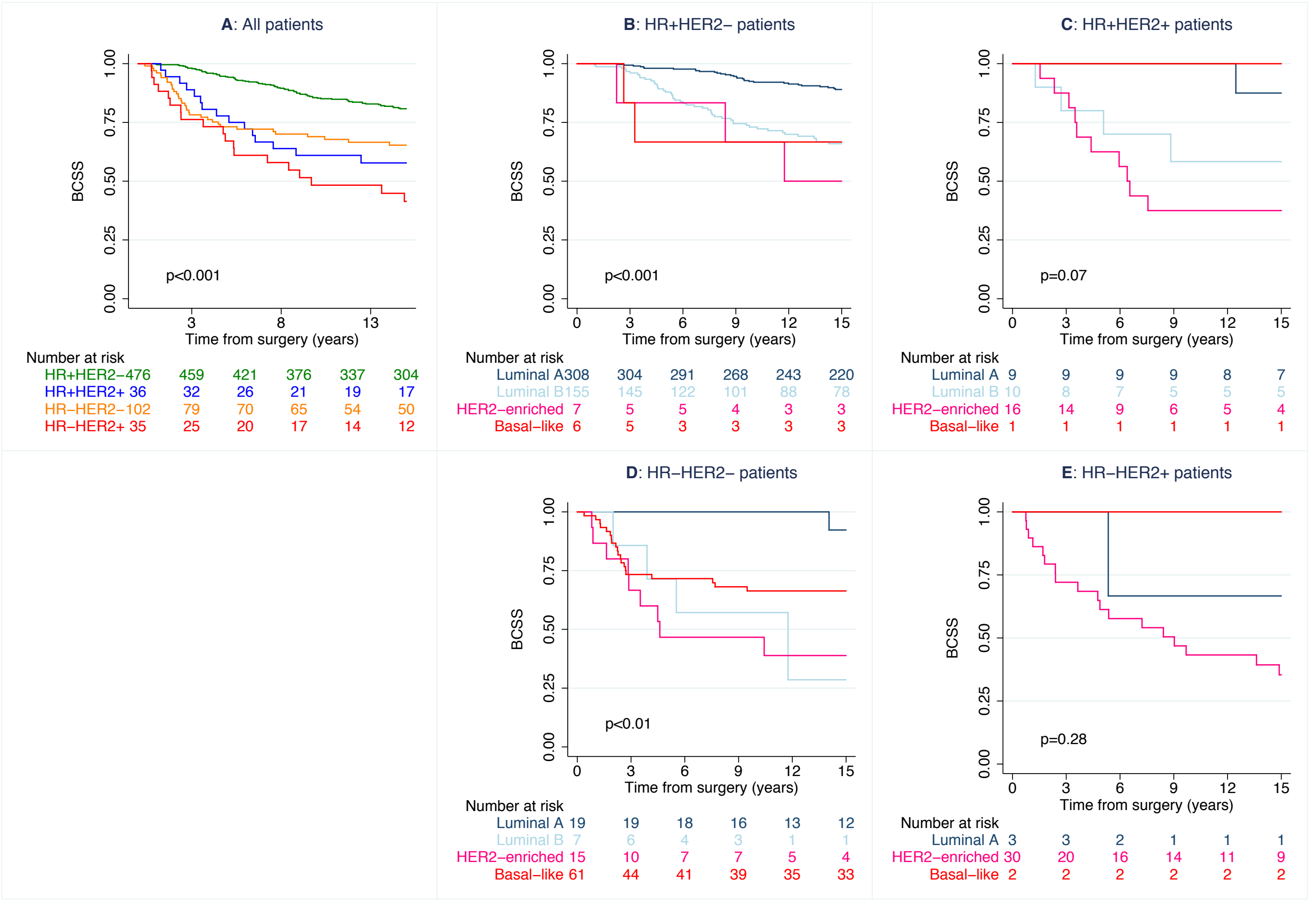
A: All patients
BCSS
1.00
0.75
0.50
0.25
0.00
p<0.001
3
8
13
Time from surgery (years)
Number at risk
HR+HER2−476 459 421 376 337 304
HR+HER2+ 36 32 26 21 19 17
HR−HER2−102 79 70 65 54 50
HR−HER2+ 35 25 20 17 14 12
B: HR+HER2− patients
BCSS
p<0.001
0 3 6 9 12 15
Time from surgery (years)
Number at risk
Luminal A308 304 291 268 243 220
Luminal B155 145 122 101 88 78
HER2−enriched 7 5 5 4 3 3
Basal−like 6 5 3 3 3 3
C: HR+HER2+ patients
BCSS
p=0.07
0 3 6 9 12 15
Time from surgery (years)
Number at risk
Luminal A 9 9 9 9 8 7
Luminal B 10 8 7 5 5 5
HER2−enriched 16 14 9 6 5 4
Basal−like 1 1 1 1 1 1
D: HR−HER2− patients
BCSS
p<0.01
0 3 6 9 12 15
Time from surgery (years)
Number at risk
Luminal A 19 19 18 16 13 12
Luminal B 7 6 4 3 1 1
HER2−enriched 15 10 7 7 5 4
Basal−like 61 44 41 39 35 33
E: HR−HER2+ patients
BCSS
p=0.28
0 3 6 9 12 15
Time from surgery (years)
Number at risk
Luminal A 3 3 2 1 1 1
HER2−enriched 30 20 16 14 11 9
Basal−like 2 2 2 2 2 2
