# Supplementary material for: Prognostic value of PAM50 and risk of recurrence score in patients with early-stage breast cancer with long-term follow-up
Source: Breast Cancer Res. 2017 Nov 14;19:120. doi: 10.1186/s13058-017-0911-9 (PMC5686844; doi:10.1186/s13058-017-0911-9)
Supplement: Supplementary file 1 — Methods. Immunohistochemical analysis for Ki-67. Figure S1. Distribution of PAM50 subtypes within subgroups based on HR and HER2 status. Bars represent percentage of total in each HR/HER2− group. Number is displayed on top of the bar. Figure S2. ROR score within each of the PAM50 subtypes for all patients (R statistical software package). Figure S3. Kaplan-Meier plots of BCSS (S3a) and DDFS (S3b) according to HR/HER2 subtypes in all 653 patients (a) and according to PAM50 subtypes within different HR/HER2 (b–e) subgroups. p Values were derived from log-rank tests. Figure S4. Kaplan-Meier plots of BCSS according to ROR categories for node-negative (a) and node-positive (b) HR+/HER2− patients. p Values were derived from log-rank tests. Figure S5. Kaplan-Meier plots of BCSS (a and b) and DDFS (c and d) according to ROR categories for node-negative luminal A HR+/HER2− patients with no adjuvant treatment (a, c) or treated with tamoxifen only (b, d). p Values were derived from log-rank tests. Figure S6. Correlation between Ki-67 expression and ROR score for the HR+/HER2− patients. ρ = 0.62, p < 0.001 (Pearson correlation). (ZIP 340 kb) [file 13058_2017_911_MOESM1_ESM.zip › Figure S3b_BCR2.1.pdf]

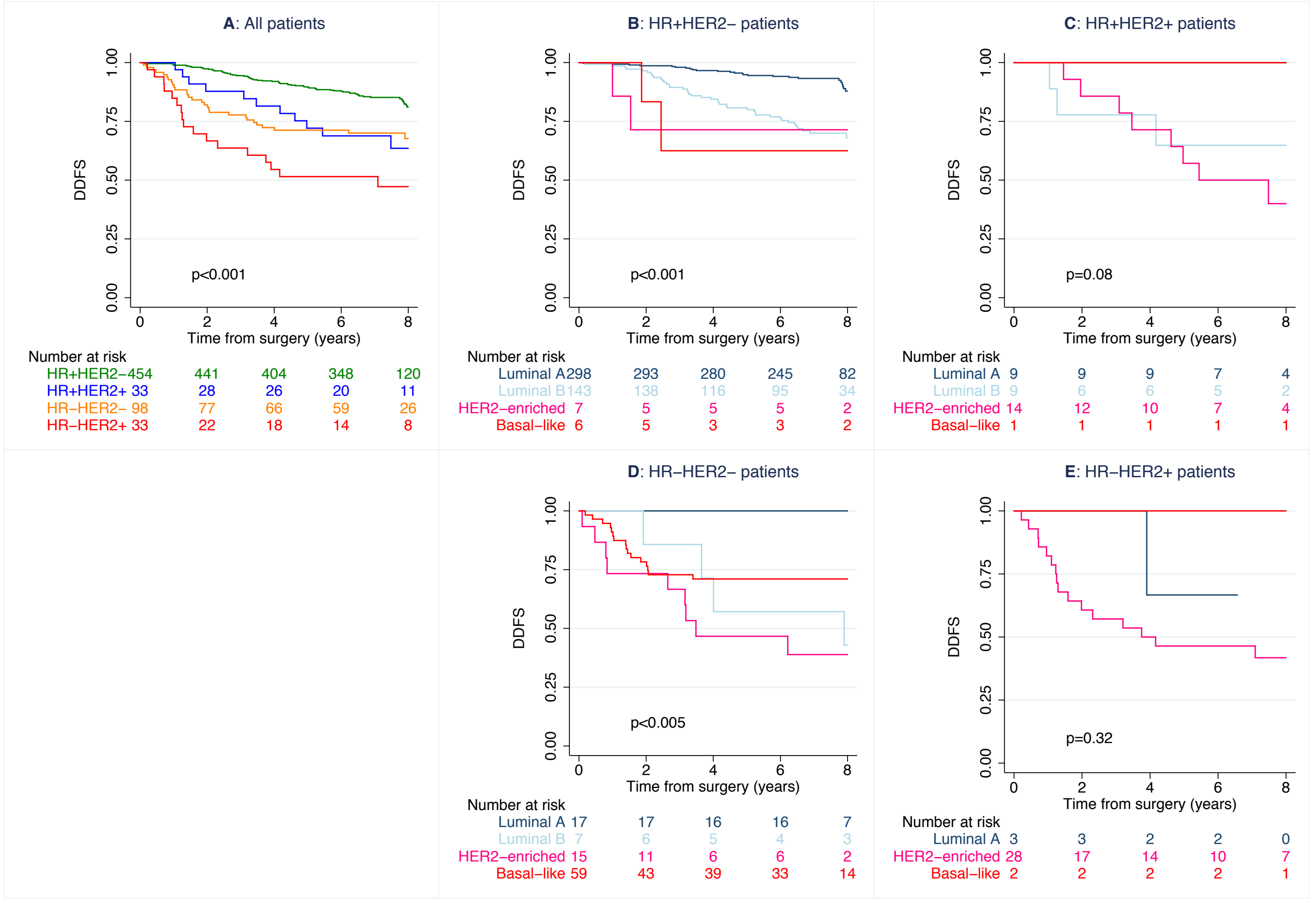
A: All patients
DDFS
1.00 0.75 0.50 0.25 0.00
p<0.001
0 2 4 6 8
Time from surgery (years)
Number at risk
HR+HER2−454 441 404 348 120
HR+HER2+ 33 28 26 20 11
HR−HER2− 98 77 66 59 26
HR−HER2+ 33 22 18 14 8
B: HR+HER2− patients
DDFS
p<0.001
Time from surgery (years)
Number at risk
Luminal A298 293 280 245 82
Luminal B143 138 116 95 34
HER2−enriched 7 5 5 5 2
Basal−like 6 5 3 3 2
C: HR+HER2+ patients
DDFS
p=0.08
Time from surgery (years)
Number at risk
Luminal A 9 9 9 7 4
Luminal B 9 6 6 5 2
HER2−enriched 14 12 10 7 4
Basal−like 1 1 1 1 1
D: HR−HER2− patients
DDFS
p<0.005
Time from surgery (years)
Number at risk
Luminal A 17 17 16 16 7
Luminal B 7 6 5 4 3
HER2−enriched 15 11 6 6 2
Basal−like 59 43 39 33 14
E: HR−HER2+ patients
DDFS
p=0.32
Time from surgery (years)
Number at risk
Luminal A 3 3 2 2 0
HER2−enriched 28 17 14 10 7
Basal−like 2 2 2 2 1
