# Supplementary material for: Prognostic value of PAM50 and risk of recurrence score in patients with early-stage breast cancer with long-term follow-up
Source: Breast Cancer Res. 2017 Nov 14;19:120. doi: 10.1186/s13058-017-0911-9 (PMC5686844; doi:10.1186/s13058-017-0911-9)
Supplement: Supplementary file 1 — Methods. Immunohistochemical analysis for Ki-67. Figure S1. Distribution of PAM50 subtypes within subgroups based on HR and HER2 status. Bars represent percentage of total in each HR/HER2− group. Number is displayed on top of the bar. Figure S2. ROR score within each of the PAM50 subtypes for all patients (R statistical software package). Figure S3. Kaplan-Meier plots of BCSS (S3a) and DDFS (S3b) according to HR/HER2 subtypes in all 653 patients (a) and according to PAM50 subtypes within different HR/HER2 (b–e) subgroups. p Values were derived from log-rank tests. Figure S4. Kaplan-Meier plots of BCSS according to ROR categories for node-negative (a) and node-positive (b) HR+/HER2− patients. p Values were derived from log-rank tests. Figure S5. Kaplan-Meier plots of BCSS (a and b) and DDFS (c and d) according to ROR categories for node-negative luminal A HR+/HER2− patients with no adjuvant treatment (a, c) or treated with tamoxifen only (b, d). p Values were derived from log-rank tests. Figure S6. Correlation between Ki-67 expression and ROR score for the HR+/HER2− patients. ρ = 0.62, p < 0.001 (Pearson correlation). (ZIP 340 kb) [file 13058_2017_911_MOESM1_ESM.zip › Figure S5_BCR2.1..pdf]

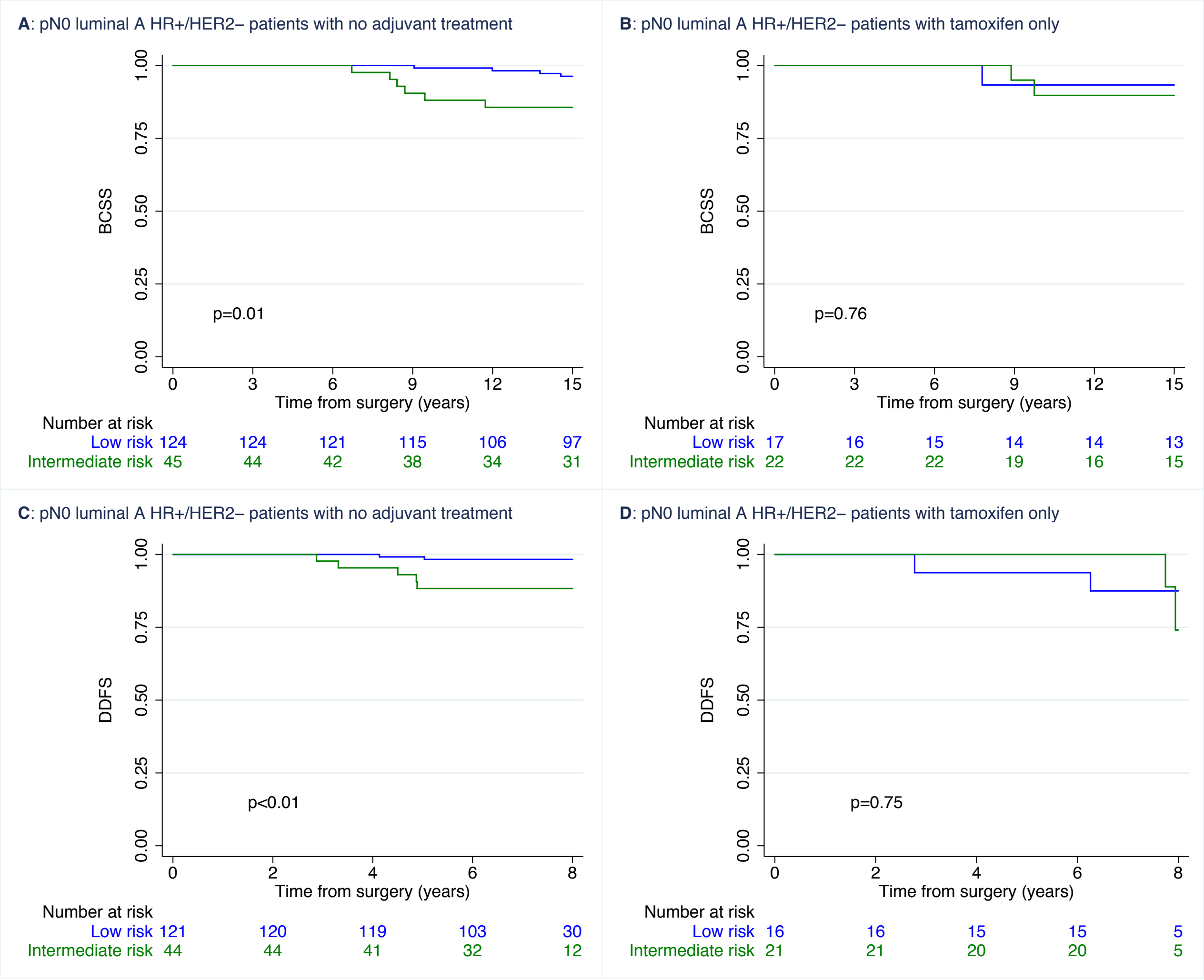
A: pN0 luminal A HR+/HER2– patients with no adjuvant treatment
BCSS
1.00
0.75
0.50
0.25
0.00
p=0.01
0
3
6
9
12
15
Time from surgery (years)
Number at risk
Low risk 124 124 121 115 106 97
Intermediate risk 45 44 42 38 34 31
B: pN0 luminal A HR+/HER2– patients with tamoxifen only
BCSS
p=0.76
Time from surgery (years)
Number at risk
Low risk 17 16 15 14 14 13
Intermediate risk 22 22 22 19 16 15
C: pN0 luminal A HR+/HER2– patients with no adjuvant treatment
DDFS
p<0.01
0
2
4
6
8
Time from surgery (years)
Number at risk
Low risk 121 120 119 103 30
Intermediate risk 44 44 41 32 12
D: pN0 luminal A HR+/HER2– patients with tamoxifen only
DDFS
p=0.75
Time from surgery (years)
Number at risk
Low risk 16 16 15 15 5
Intermediate risk 21 21 20 20 5
