# Supplementary material for: Prognostic value of PAM50 and risk of recurrence score in patients with early-stage breast cancer with long-term follow-up
Source: Breast Cancer Res. 2017 Nov 14;19:120. doi: 10.1186/s13058-017-0911-9 (PMC5686844; doi:10.1186/s13058-017-0911-9)
Supplement: Supplementary file 1 — Methods. Immunohistochemical analysis for Ki-67. Figure S1. Distribution of PAM50 subtypes within subgroups based on HR and HER2 status. Bars represent percentage of total in each HR/HER2− group. Number is displayed on top of the bar. Figure S2. ROR score within each of the PAM50 subtypes for all patients (R statistical software package). Figure S3. Kaplan-Meier plots of BCSS (S3a) and DDFS (S3b) according to HR/HER2 subtypes in all 653 patients (a) and according to PAM50 subtypes within different HR/HER2 (b–e) subgroups. p Values were derived from log-rank tests. Figure S4. Kaplan-Meier plots of BCSS according to ROR categories for node-negative (a) and node-positive (b) HR+/HER2− patients. p Values were derived from log-rank tests. Figure S5. Kaplan-Meier plots of BCSS (a and b) and DDFS (c and d) according to ROR categories for node-negative luminal A HR+/HER2− patients with no adjuvant treatment (a, c) or treated with tamoxifen only (b, d). p Values were derived from log-rank tests. Figure S6. Correlation between Ki-67 expression and ROR score for the HR+/HER2− patients. ρ = 0.62, p < 0.001 (Pearson correlation). (ZIP 340 kb) [file 13058_2017_911_MOESM1_ESM.zip › Supplementary material_BCR2.1.docx]

**Supplementary material**

**Methods**

**Immunohistochemical analysis for Ki67**

One whole section from a FFPE block from the primary tumour was immunostained for Ki67 with clone MIB1( Dako M7240) in dilution 1:100, on a BenchMark ULTRA staining system, using CC1 standard antigen retrieval and UltraView DAB with amplification as detection system. Sensitivity/specificity of the staining protocol was adapted to satisfy the recommendations from NordiQC (<http://www.nordiqc.org/>), and tonsil was used as positive control. The slides were screened manually in a light microscope to identify the area with the highest Ki67 tumor cell expression. Then, using the 40 lens, the proportion of tumor cells with a Ki67-positive nucleus was counted in the selected area, helped by using an ocular provided with a 10x10 graticule (Pyser-SGI). At least 250 tumor cell nuclei were analyzed, and the proportion of Ki67-positive tumor cells was registered (“hot-spot” score).

**Supplementary figure legends**

**Figure S1**

Distribution of PAM50 subtypes within subgroups based on HR and HER2 status. Bars representing percent of total in each HR/HER2-group, “N” displayed on top of the bar.

**Figure S2**

ROR score within each of the PAM50 subtypes for all patients (Statistical package “R”)

**Figure S3**

Kaplan–Meier plots of BCSS (3a) and DDFS (3b) according to HR/HER2 subtypes in all 653 patients (A) and according to PAM50 subtypes within different HR/HER2 (B-E) subgroups. P-values from Log-rank tests.

**Figure S4**

Kaplan–Meier plots of BCSS according to ROR categories for node negative (A) and node positive (B) HR+/HER2- patients . P-values from Log-rank tests.

**Figure S5**

Kaplan-Meier plots of BCSS (A-B) and DDFS (C-D) according to ROR categories for node negative luminal A HR+/HER2- patients with no adjuvant treatment (A, C) or treated with tamoxifen only (B, D). P-values from Log-rank tests.

**Figure S6**

Correlation between Ki67 expression and ROR score for the HR+/HER2- patients. rho= 0.62, *p<0.001*(Pearson correlation)

**Comment to supplementary Table S1**

The PAM50 subtype classification was a strong prognostic factor in addition to the established clinicopathological variables for all patients. Patients with luminal A tumors had improved BCSS compared to those with luminal B (HR 3.04, 95% CI: 1.93-4.80), HER2 enriched (HR 4.13, 95% CI: 2.09-8.17) and basal-like tumors (HR 2.30, 95% CI: 1.01-5.23). Similar prognostic information was observed for DDFS.

**Comments to Figures**

**Supplementary Figure S1**

The majority of the tumors categorized as HR+/HER2- by immunohistochemistry were of the luminal A or B subtypes (94 %) and most of the HR-/HER2+ were HER2 enriched (86%). Among the HR-/HER2- tumors, 60% were of basal-like subtype. The HR+/HER2+ subgroup consisted of both luminal A/B and HER2 enriched subtypes.

**Supplementary Figure S5**

Comment: Among HR+/HER2- and HR-/HER2- patients, those with luminal A tumors had a low risk of distant disease and breast cancer death, whereas patients with luminal B, basal-like and HER2 enriched tumors had high risk of distant disease and breast cancer death
