# Supplementary figures and images for: Prognostic value of PAM50 and risk of recurrence score in patients with early-stage breast cancer with long-term follow-up
Source: Breast Cancer Res. 2017 Nov 14;19:120. doi: 10.1186/s13058-017-0911-9 (PMC5686844; doi:10.1186/s13058-017-0911-9)

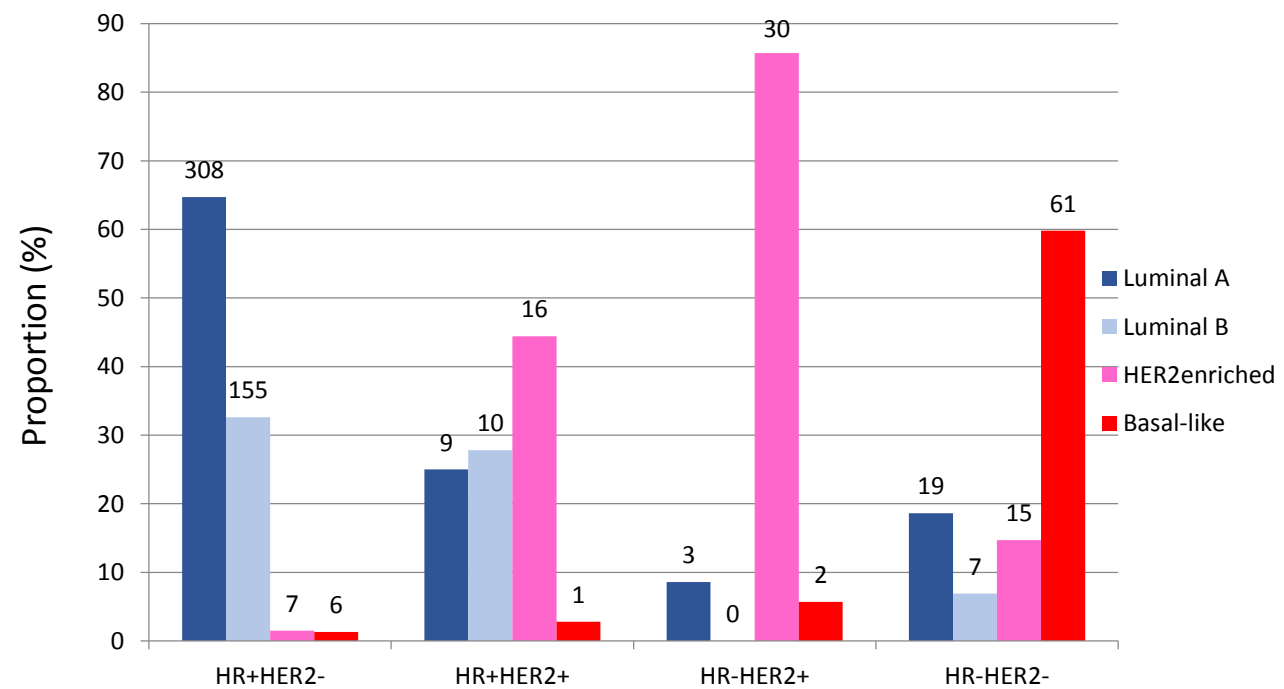

Supplement: Supplementary file 1 — Methods. Immunohistochemical analysis for Ki-67. Figure S1. Distribution of PAM50 subtypes within subgroups based on HR and HER2 status. Bars represent percentage of total in each HR/HER2− group. Number is displayed on top of the bar. Figure S2. ROR score within each of the PAM50 subtypes for all patients (R statistical software package). Figure S3. Kaplan-Meier plots of BCSS (S3a) and DDFS (S3b) according to HR/HER2 subtypes in all 653 patients (a) and according to PAM50 subtypes within different HR/HER2 (b–e) subgroups. p Values were derived from log-rank tests. Figure S4. Kaplan-Meier plots of BCSS according to ROR categories for node-negative (a) and node-positive (b) HR+/HER2− patients. p Values were derived from log-rank tests. Figure S5. Kaplan-Meier plots of BCSS (a and b) and DDFS (c and d) according to ROR categories for node-negative luminal A HR+/HER2− patients with no adjuvant treatment (a, c) or treated with tamoxifen only (b, d). p Values were derived from log-rank tests. Figure S6. Correlation between Ki-67 expression and ROR score for the HR+/HER2− patients. ρ = 0.62, p < 0.001 (Pearson correlation). (ZIP 340 kb) [file 13058_2017_911_MOESM1_ESM.zip › Figure S1_BCR2.1.pdf]

ROR

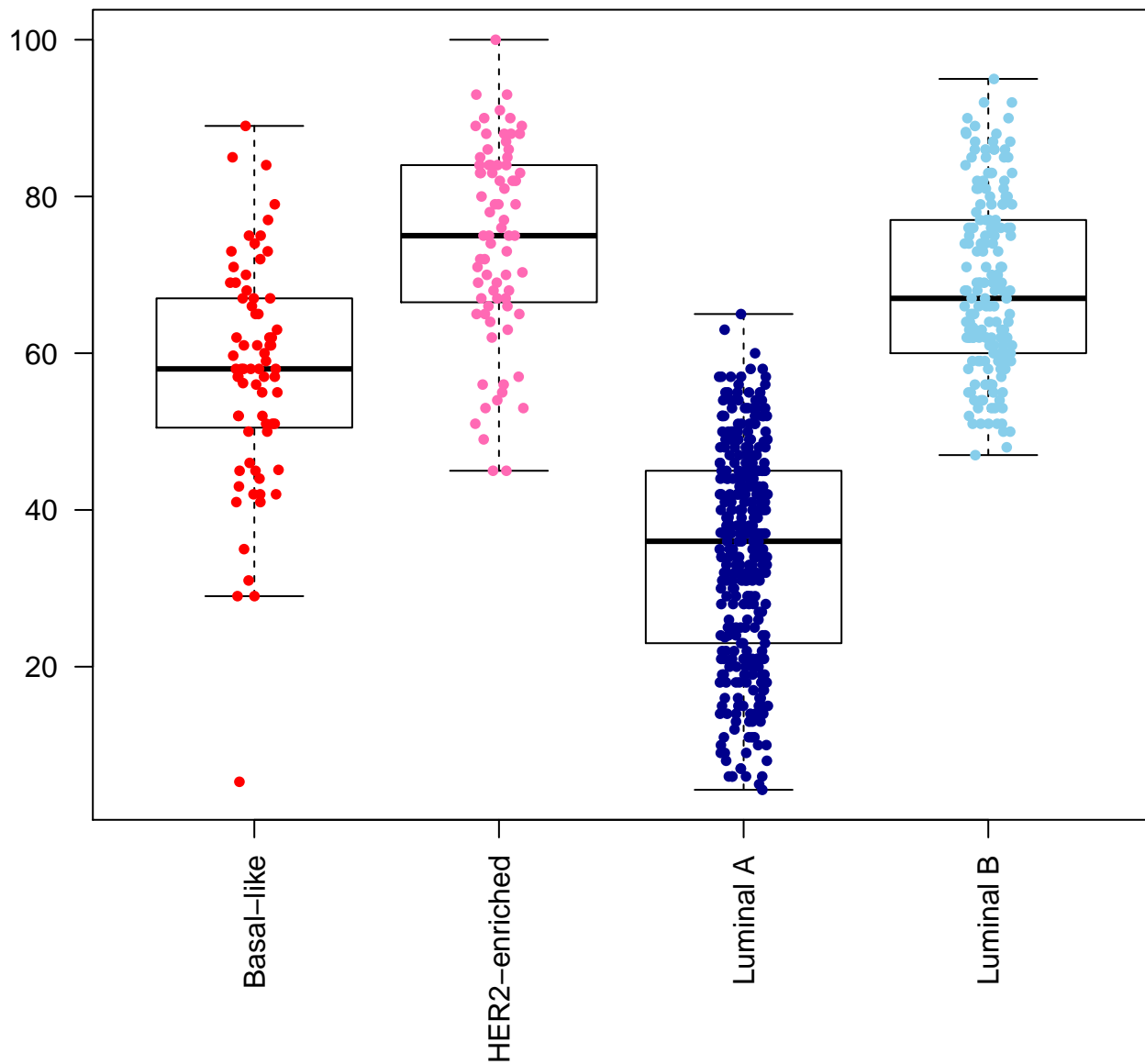

Supplement: Supplementary file 1 — Methods. Immunohistochemical analysis for Ki-67. Figure S1. Distribution of PAM50 subtypes within subgroups based on HR and HER2 status. Bars represent percentage of total in each HR/HER2− group. Number is displayed on top of the bar. Figure S2. ROR score within each of the PAM50 subtypes for all patients (R statistical software package). Figure S3. Kaplan-Meier plots of BCSS (S3a) and DDFS (S3b) according to HR/HER2 subtypes in all 653 patients (a) and according to PAM50 subtypes within different HR/HER2 (b–e) subgroups. p Values were derived from log-rank tests. Figure S4. Kaplan-Meier plots of BCSS according to ROR categories for node-negative (a) and node-positive (b) HR+/HER2− patients. p Values were derived from log-rank tests. Figure S5. Kaplan-Meier plots of BCSS (a and b) and DDFS (c and d) according to ROR categories for node-negative luminal A HR+/HER2− patients with no adjuvant treatment (a, c) or treated with tamoxifen only (b, d). p Values were derived from log-rank tests. Figure S6. Correlation between Ki-67 expression and ROR score for the HR+/HER2− patients. ρ = 0.62, p < 0.001 (Pearson correlation). (ZIP 340 kb) [file 13058_2017_911_MOESM1_ESM.zip › Figure S2_BCR2.1.pdf]

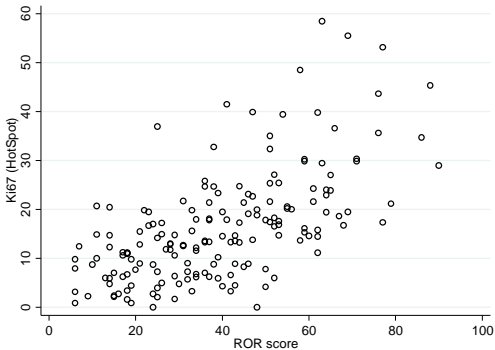

Supplement: Supplementary file 1 — Methods. Immunohistochemical analysis for Ki-67. Figure S1. Distribution of PAM50 subtypes within subgroups based on HR and HER2 status. Bars represent percentage of total in each HR/HER2− group. Number is displayed on top of the bar. Figure S2. ROR score within each of the PAM50 subtypes for all patients (R statistical software package). Figure S3. Kaplan-Meier plots of BCSS (S3a) and DDFS (S3b) according to HR/HER2 subtypes in all 653 patients (a) and according to PAM50 subtypes within different HR/HER2 (b–e) subgroups. p Values were derived from log-rank tests. Figure S4. Kaplan-Meier plots of BCSS according to ROR categories for node-negative (a) and node-positive (b) HR+/HER2− patients. p Values were derived from log-rank tests. Figure S5. Kaplan-Meier plots of BCSS (a and b) and DDFS (c and d) according to ROR categories for node-negative luminal A HR+/HER2− patients with no adjuvant treatment (a, c) or treated with tamoxifen only (b, d). p Values were derived from log-rank tests. Figure S6. Correlation between Ki-67 expression and ROR score for the HR+/HER2− patients. ρ = 0.62, p < 0.001 (Pearson correlation). (ZIP 340 kb) [file 13058_2017_911_MOESM1_ESM.zip › Figure S6_BCR2.1.pdf]
