# Supplementary material for: Prognostic value of PAM50 and risk of recurrence score in patients with early-stage breast cancer with long-term follow-up
Source: Breast Cancer Res. 2017 Nov 14;19:120. doi: 10.1186/s13058-017-0911-9 (PMC5686844; doi:10.1186/s13058-017-0911-9)
Supplement: Supplementary file 2 — Multivariate analysis of DDFS and BCSS for all patients, including PAM50 intrinsic subtypes. (DOC 49 kb) [file 13058_2017_911_MOESM2_ESM.doc]

**Additional file Supplementary Table S1:** Multivariate Analysis of DDFS and BCSS for all patients including PAM50 intrinsic subtypes

|  |  | **DDFS (N=601)** | | |  | **BCSS (N=627)** | | |  |
| --- | --- | --- | --- | --- | --- | --- | --- | --- | --- |
|  |  | **HR** | **95% CI** | **p** |  | **HR** | **95% CI** | **P** | |
| **pT** | 1 | 1 |  |  |  | 1 |  |  | |
|  | 2 | 1.79 | 1.20-2.67 | 0.005 |  | 1.75 | 1.22-2.52 | 0.003 | |
|  | 3-4 | 1.60 | 0.71-3.58 | 0.253 |  | 1.66 | 0.81-3.41 | 0.169 | |
| **Grade** | I | 1 |  |  |  | 1 |  |  | |
|  | II | 1.66 | 0.79-3.49 | 0.184 |  | 1.94 | 0.97-3.88 | 0.062 | |
|  | III | 1.64 | 0.71-3.78 | 0.245 |  | 2.17 | 1.00-4.72 | 0.051 | |
| **pN** | 0 | 1 |  |  |  | 1 |  |  | |
|  | 1 | 3.60 | 2.08-6.25 | <0.001 |  | 2.61 | 1.59-4.27 | <0.001 | |
|  | 2-3 | 5.72 | 3.12-10.48 | <0.001 |  | 4.25 | 2.49-7.25 | <0.001 | |
| **Age** | < 55 years | 1 |  |  |  | 1 |  |  | |
|  | ≥ 55 years | 0.65 | 0.39-1.06 | 0.083 |  | 0.82 | 0.53-1.27 | 0.380 | |
| **Systemic treatment** | No | 1 |  |  |  | 1 |  |  | |
|  | Tamoxifen | 0.87 | 0.49-1.54 | 0.635 |  | 1.00 | 0.60-1.65 | 0.999 | |
|  | Chemo ± Tam | 0.28 | 0.14-0.57 | <0.001 |  | 0.41 | 0.22-0.76 | 0.004 | |
| **HR/HER2** | HR+HER2- | 1 |  |  |  | 1 |  |  | |
|  | HR+HER2+ | 0.81 | 0.38-1.73 | 0.586 |  | 1.13 | 0.60-2.16 | 0.700 | |
|  | HR-HER2+ | 1.54 | 0.82-2.88 | 0.176 |  | 1.64 | 0.90-3.00 | 0.106 | |
|  | HR-HER2- | 2.19 | 1.00-4.77 | 0.049 |  | 2.07 | 1.01-4.24 | 0.048 | |
| **PAM50 intrinsic subtype** | Luminal A | 1 |  |  |  | 1 |  |  | |
|  | Luminal B | 3.58 | 2.14-5.99 | <0.001 |  | 3.04 | 1.93-4.80 | <0.001 | |
|  | HER2enriched | 5.23 | 2.47-11.06 | <0.001 |  | 4.13 | 2.09-8.17 | <0.001 | |
|  | Basal-like | 3.25 | 1.35-7.84 | 0.009 |  | 2.30 | 1.01-5.23 | 0.047 | |
